# Supplementary material for: HIVprotI: an integrated web based platform for prediction and design of HIV proteins inhibitors
Source: J Cheminform. 2018 Mar 9;10:12. doi: 10.1186/s13321-018-0266-y (PMC5845081; doi:10.1186/s13321-018-0266-y)
Supplement: Supplementary file 2 — Additional file 2. Source code of HIVProtI web server. [file 13321_2018_266_MOESM2_ESM.zip › HIVprotI_Source-code/hivprotI/weka-3-6-6/doc/weka/filters/unsupervised/attribute/StringToNominal.html]

StringToNominal


---


|  |  |  |
| --- | --- | --- |
| **Overview** \| **Package** \| **Class** \| **Tree** \| **Deprecated** \| **Index** \| **Help** \| **Weka's home** | |  |
| **PREV CLASS**   **NEXT CLASS** | **FRAMES**    **NO FRAMES**     **All Classes** |  |
| SUMMARY: NESTED \| FIELD \| CONSTR \| METHOD | DETAIL: FIELD \| CONSTR \| METHOD |  |


---


## weka.filters.unsupervised.attribute Class StringToNominal

```
java.lang.Object
  weka.filters.Filter
      weka.filters.unsupervised.attribute.StringToNominal
```

**All Implemented Interfaces:**: java.io.Serializable, CapabilitiesHandler, OptionHandler, RevisionHandler, UnsupervisedFilter

---

``` public class StringToNominal extends Filter implements UnsupervisedFilter, OptionHandler ```

Converts a string attribute (i.e. unspecified number of values) to nominal (i.e. set number of values). You should ensure that all string values that will appear are represented in the first batch of the data.


Valid options are:

```
 -R <col>
  Sets the range of attribute indices (default last).
```

**Version:**
:   $Revision: 7103 $

**Author:**
:   Len Trigg (len@reeltwo.com)

**See Also:**: Serialized Form

---

**Constructor Summary**

`StringToNominal()`


| **Method Summary** | |
| --- | --- |
| `java.lang.String` | `attributeRangeTipText()` |
| `boolean` | `batchFinished()`             Signifies that this batch of input to the filter is finished. |
| `java.lang.String` | `getAttributeRange()`             Get the range of indices of the attributes used. |
| `Capabilities` | `getCapabilities()`             Returns the Capabilities of this filter. |
| `java.lang.String[]` | `getOptions()`             Gets the current settings of the filter. |
| `java.lang.String` | `getRevision()`             Returns the revision string. |
| `java.lang.String` | `globalInfo()`             Returns a string describing this filter |
| `boolean` | `input(Instance instance)`             Input an instance for filtering. |
| `java.util.Enumeration<Option>` | `listOptions()`             Returns an enumeration describing the available options. |
| `static void` | `main(java.lang.String[] argv)`             Main method for testing this class. |
| `void` | `setAttributeRange(java.lang.String rangeList)`             Sets range of indices of the attributes used. |
| `boolean` | `setInputFormat(Instances instanceInfo)`             Sets the format of the input instances. |
| `void` | `setOptions(java.lang.String[] options)`             Parses a given list of options. |

**Methods inherited from class weka.filters.Filter**

`batchFilterFile, filterFile, getCapabilities, getOutputFormat, isFirstBatchDone, isNewBatch, isOutputFormatDefined, makeCopies, makeCopy, numPendingOutput, output, outputPeek, toString, useFilter, wekaStaticWrapper`

**Methods inherited from class java.lang.Object**

`equals, getClass, hashCode, notify, notifyAll, wait, wait, wait`

**Constructor Detail**

### StringToNominal

```
public StringToNominal()
```


**Method Detail**

### globalInfo

```
public java.lang.String globalInfo()
```

:   Returns a string describing this filter

    :   **Returns:**: a description of the filter suitable for displaying in the explorer/experimenter gui

---


### getCapabilities

```
public Capabilities getCapabilities()
```

:   Returns the Capabilities of this filter.

    :   **Specified by:**: `getCapabilities` in interface `CapabilitiesHandler` **Overrides:**: `getCapabilities` in class `Filter`
    :   **Returns:**: the capabilities of this object **See Also:**: `Capabilities`

---


### setInputFormat

```
public boolean setInputFormat(Instances instanceInfo)
                       throws java.lang.Exception
```

:   Sets the format of the input instances.

    :   **Overrides:**: `setInputFormat` in class `Filter`
    :   **Parameters:**: `instanceInfo` - an Instances object containing the input instance structure (any instances contained in the object are ignored - only the structure is required). **Returns:**: true if the outputFormat may be collected immediately. **Throws:**: `UnsupportedAttributeTypeException` - if the selected attribute a string attribute.: `java.lang.Exception` - if the input format can't be set successfully.

---


### input

```
public boolean input(Instance instance)
```

:   Input an instance for filtering. The instance is processed
    and made available for output immediately.

    :   **Overrides:**: `input` in class `Filter`
    :   **Parameters:**: `instance` - the input instance. **Returns:**: true if the filtered instance may now be collected with output(). **Throws:**: `java.lang.IllegalStateException` - if no input structure has been defined.

---


### batchFinished

```
public boolean batchFinished()
```

:   Signifies that this batch of input to the filter is finished. If the
    filter requires all instances prior to filtering, output() may now
    be called to retrieve the filtered instances.

    :   **Overrides:**: `batchFinished` in class `Filter`
    :   **Returns:**: true if there are instances pending output. **Throws:**: `java.lang.IllegalStateException` - if no input structure has been defined.

---


### listOptions

```
public java.util.Enumeration<Option> listOptions()
```

:   Returns an enumeration describing the available options.

    :   **Specified by:**: `listOptions` in interface `OptionHandler`
    :   **Returns:**: an enumeration of all the available options.

---


### setOptions

```
public void setOptions(java.lang.String[] options)
                throws java.lang.Exception
```

:   Parses a given list of options.


    Valid options are:

    ```
     -R <col>
      Sets the range of attribute indices (default last).
    ```

    ```
     -V <col>
      Inverts the selection specified by -R.
    ```

    :   **Specified by:**: `setOptions` in interface `OptionHandler`
    :   **Parameters:**: `options` - the list of options as an array of strings **Throws:**: `java.lang.Exception` - if an option is not supported

---


### getOptions

```
public java.lang.String[] getOptions()
```

:   Gets the current settings of the filter.

    :   **Specified by:**: `getOptions` in interface `OptionHandler`
    :   **Returns:**: an array of strings suitable for passing to setOptions

---


### attributeRangeTipText

```
public java.lang.String attributeRangeTipText()
```

:   **Returns:**: tip text for this property suitable for displaying in the explorer/experimenter gui

---


### getAttributeRange

```
public java.lang.String getAttributeRange()
```

:   Get the range of indices of the attributes used.

    :   **Returns:**: the index of the attribute

---


### setAttributeRange

```
public void setAttributeRange(java.lang.String rangeList)
```

:   Sets range of indices of the attributes used.

    :   **Parameters:**: `attIndex` - the index of the attribute

---


### getRevision

```
public java.lang.String getRevision()
```

:   Returns the revision string.

    :   **Specified by:**: `getRevision` in interface `RevisionHandler` **Overrides:**: `getRevision` in class `Filter`
    :   **Returns:**: the revision

---


### main

```
public static void main(java.lang.String[] argv)
```

:   Main method for testing this class.

    :   **Parameters:**: `argv` - should contain arguments to the filter: use -h for help


---


|  |  |  |
| --- | --- | --- |
| **Overview** \| **Package** \| **Class** \| **Tree** \| **Deprecated** \| **Index** \| **Help** \| **Weka's home** | |  |
| **PREV CLASS**   **NEXT CLASS** | **FRAMES**    **NO FRAMES**     **All Classes** |  |
| SUMMARY: NESTED \| FIELD \| CONSTR \| METHOD | DETAIL: FIELD \| CONSTR \| METHOD |  |


---
